# Disinfectants and Their Application to Therapeutics

**Published:** 1863-08

**Authors:** 


### DISINFECTANTS AND THEIR APPLICATION TO THERAPEUTICS.

Conclusions from facts contained in a memoir upon the subject published in the *Archives Générales*, by O. Reveil, Professeur agrégé à la Faculté de Médecine et à l'école superieure de Pharmacie, &c., &c.

1st, That there probably exist many kinds of putrid fermentations, varying in their causes as in their effects.

2d, That there is no general disinfectant capable of being indiscriminately used in all cases;

3d, That liquid disinfectants are always preferable to others, other things being equal, when applied in therapeutics.

In their application to this purpose regard should be paid to their cost, the facility of their employment and the inconveniences they may cause by corroding, soiling or rendering unserviceable the linen dressings.

4th, The best disinfectant is that which possesses the following properties:—it should—A. Instantly destroy or mask bad odors; B. Absorb the liquid or gaseous products of the putrefactive or inflammatory process, remove them by washing and destroy the poisonous or irritating action of morbid liquids and mephitic gaseous products; C. Prevent the formation of new infections or mephitic products; D. Hasten the cicatrization of sores, by giving the necessary vitality for the reparation of the tissues.

5th, Chlorine, and solutions of bromine and iodine, appear to best fulfill the most important of these conditions.

6th, Chlorine, or at least the hypochlorites, by reason of the gaseous state of their active principle, ought always to be preferred when it is desired to destroy miasma and disinfect the air.

7th, The addition of odorous essences, and principally of nitro-benzine to the hypochlorites and to iodine and bromine-water, acts both to mask the disagreeable odors and to set into immediate operation the chemical action.

8th, Tar and coal-tar preparations are able to render effectual service, but they do not possess the property, like iodine and bromine, of destroying the poisonous action of morbid products and putrefaction, or that of various kinds of virus.

9th, *Charpie carbonifere*, and especially *charpie carbonifere iodée*, may be often employed with success.

10th, Carbon, in addition to its absorbant properties, appears to exercise *an action of special contact*, in virtue of which it hastens the destruction of organic matters, or rather, as M. Stenhouse states, according to the experience of Turnbull and Turner, by condensing the oxygen of the air, and thus acting as spongy platinum.

11th, Metallic solutions (salts of iron, zinc, &c.), although imperfect disinfectants, suffice in a great number of cases.

12th, Physical and mechanical agents (ventilators, &c.) may be made powerful aids to chemical disinfectants.

13th, There are some causes of *infection* which appear to resist all treatment (ozæna, otitis, &c.)

14th, We should add, moreover, that there are causes of *infection* which it would be dangerous to suppress (*sueur infecte des pieds*), and the odor of which we should endeavor to mask.

One is struck with admiration on reflecting upon the processes which nature employs to disseminate, transform and reproduce organic matters; in the presence of the grandeur of these facts, we remain convinced of the exactitude of the aphorism of Lavoisier:—" *Dans la nature, rien ne se perd, rien ne se crée.*"

M. Reveil alludes above to the so-called fœtid foot-sweat, and thus sustains a popular error both ofpathology and therapeutics. Fœtid foot-sweat does not exist. The perspiration is always fresh when secreted, and any peculiar individual odor is due to specific modifications in the sebaceous matter. It is only when the sweat has undergone decomposition upon certain portions of the body peculiarly adapted to such change, as the axillæ, beneath the mammæ, between the folds of the prinæum, &c., that the fatty acids thus formed give rise to a fœtid odor. So, too, when the feet are not frequently cleansed, and heavy coverings are worn upon them, the boot is apt to absorb the odors thus produced, and to become in turn the real offender. We have noticed this peculiarly disagreeable smell about the persons of several officers returning from the army, where thick stockings and heavy-top boots are often worn day and night without change for a considerable time. The idea of any dangerous consequences following simple cleanliness, which is the only disinfectant required, is absurd. The worst case of fœtid foot sweat may be cured at once by casting away the stinking boots, and substituting thin stockings and light boots or shoes, and by washing the feet every day in soap and water.

